# Association between maternal HIV infection and low birth weight and prematurity: a meta-analysis of cohort studies

**DOI:** 10.1186/s12884-015-0684-z

**Published:** 2015-10-08

**Authors:** Peng-Lei Xiao, Yi-Biao Zhou, Yue Chen, Mei-Xia Yang, Xiu-Xia Song, Yan Shi, Qing-Wu Jiang

**Affiliations:** 1 Fudan University School of Public Health, Building 8, 130 Dong’an Road, Xuhui District, Shanghai, 200032 China; 2 Key Laboratory of Public Health Safety, Fudan University, Ministry of Education, Building 8, 130 Dong’an Road, Xuhui District, Shanghai, 200032 China; 3 Fudan University Center for Tropical Disease Research, Building 8, 130 Dong’an Road, Xuhui District, Shanghai, 200032 China; 4 School of Epidemiology, Public Health and Preventive Medicine, Faculty of Medicine, University of Ottawa, 451 Smyth Road, Ottawa, ON K1H 8 M5 Canada; 5 Xuhui Center for Disease Prevention and Control, 50 Yongchuan Road, Xuhui District, Shanghai, 200032 China

**Keywords:** Maternal HIV infection, Meta-analysis, Low birth weight, Preterm delivery

## Abstract

**Background:**

To assess the association between maternal human immunodeficiency virus (HIV) infection and low birth weight (LBW)/prematurity (PTD), we conducted a meta-analysis of cohort studies of HIV infected and uninfected women.

**Methods:**

Several English and Chinese databases were searched (updated to May 2015) to find the studies reporting infant outcomes associated with exposure to maternal HIV infection during pregnancy. Relevant articles were manually selected based on several inclusion and exclusion criteria.

**Results:**

Fifty-two cohort studies including 15,538 (for LBW) and 200,896 (for PTD) HIV infected women met the inclusion criteria. There was significant heterogeneity among studies for maternal HIV infection associated with LBW/PTD (I^2^ = 71.7 %, *P* < 0.05, and I^2^ = 51.8 %, *P* < 0.05 for LBW and PTD, respectively). The meta-analysis demonstrated that the maternal HIV infection was significantly associated with both LBW (pooled odds ratio (OR): 1.73, 95 % confidence interval (CI): 1.64, 1.82, *P* < 0.001) and PTD (pooled OR: 1.56, 95 % CI: 1.49, 1.63, *P* < 0.001). No significant difference in the relationship between maternal HIV infection and adverse pregnancy outcomes was detected among the groups of different study periods. HIV infected women were at slightly higher risk of LBW in developing countries compared with women in developed countries (OR: 2.12 (95 % CI: 1.81, 2.48) vs. 1.75 (95 % CI: 1.44, 2.12)). Antiretroviral drugs usage did not significantly change the associations of maternal HIV exposure with LBW and PTD.

**Conclusions:**

HIV infected women were at higher risk of having a low birth weight infant or a preterm delivery infant compared with uninfected women. Such associations did not change significantly over time or were not significantly affected by the usage of antiretroviral drugs.

**Electronic supplementary material:**

The online version of this article (doi:10.1186/s12884-015-0684-z) contains supplementary material, which is available to authorized users.

## Background

Although new infections of human immunodeficiency virus (HIV) show a descending trend in recent years, the number of people living with HIV has been rising year by year. More than 16 million (95 % CI: 14.8 million, 17.4 million) female adults had been infected with HIV by the end of 2012 [1]. There is a risk for mother-to-child transmission of HIV. Among HIV infected women who took the highly active antiretroviral therapy (HAART), studies reported that the mother-to-child transmission (MTCT) rate ranged from 1 to 5 % [2], and it was around 10 % among women who did not [3, 4]. There is also a possibility that maternal HIV infection has severe impacts on pregnancy outcomes. It has been reported that HIV infected women are more likely to encounter adverse pregnancy outcomes, such as low birth weight (LBW) and preterm delivery (PTD) [5]. And it is suggested that LBW and PTD are important risk factors for post-neonatal mortality and morbidity and other adverse events including neurodevelopmental problems [6, 7].

Studies have provided inconsistent results for the association between maternal HIV infection and LBW/PTD. Some studies suggested that maternal HIV infection could increase the risk of LBW and PTD [8–10], but others reported no significant association between them [11–13]. Brocklehurst et al. [5] summarized the study results published between 1983 and 1996. They found the association of maternal HIV infection with adverse pregnancy outcomes such as LBW and PTD, but failed to assess the effect of antiretroviral drugs on it. There have been many new studies on the association between maternal HIV infection and adverse pregnancy outcomes reported in the past 20 years. For this reason, we conducted a meta-analysis to provide an update on the relationship between maternal HIV infection and LBW/PTD. In addition, there has been a rapid progress of medication and heath care for HIV infected women, and we assessed the influence of such a progress on the relationship.

## Methods

### Search strategy

We searched English databases (EMBASE, MEDLINE via PubMed and Web of Science) and Chinese databases (China National Knowledge Infrastructure, Wan Fang database, Sino Med and VIP) up to May 31, 2015 for studies reporting infant outcomes among women exposed and unexposed to HIV infection during pregnancy. Articles were searched using the following search terms: “maternal HIV infection” “HIV infected mothers/HIV infected pregnant women” combined with “pregnant outcomes/birth outcomes” “low birth weight (LBW)” “prematurity/preterm birth/preterm delivery (PTD)”. Only cohort studies were included.

### Study selection

The relevant articles were manually selected based on the following criteria: (1) Types of studies: Prospective or retrospective cohort studies; unsystematic observations (case series or case reports) were excluded from all analyses. Because cohort studies demonstrate strong intensity compared with other observational studies in terms of causality. (2) Types of participants: Women with HIV infection during pregnancy. (3) Types of comparators: Women with no HIV infection during pregnancy. (4) Types of pregnancy outcomes: LBW (defined as birth weight <2500 g) and PTD (defined as gestational age <37 weeks). (5) All the articles reported or allowed the calculation of odd ratios (OR) with corresponding 95 % confidence intervals (95 % CIs). In order to standardize the results, all measures of effect were expressed as OR and 95 % CIs. (6) Published in English or Chinese, and full text was available. When the same cohort population was used in several papers, only the most recent ones were included in the current analysis, i.e., the same study data were only used once in the study.

### Assessment of study quality and data extraction

We adapted the Newcastle-Ottawa Scale suggested by Zeng et al. [14] to measure study quality. Scores were awarded for each criterion met, and the maximum score was 9. Studies were graded high quality if the score was no less than 7 and low quality if the score was under 7. Abstracts and conference papers were graded 0 for quality and were included in the low quality category for sensitivity analyses.

Two researchers selected literature in accordance with the inclusion and exclusion criteria, and extracted data after independent verification. The following information was extracted from each study: subject of the literature, the author, year of publication,location of the study population, number of women in the two groups, odds ratio and its 95 % CIs for LBW and PTD associated with maternal HIV infection, exposure to maternal use of antiretroviral drugs before or during pregnancy.

### Statistical analysis

Stata12.0 software was used to analyze the data. For both LBW and PTD, if no significant heterogeneity for their associations with maternal HIV exposure among the studies included was found (*P* > 0.1, *I*^*2*^ < 50 %), a fixed effect model was used to calculate ORs and their 95 % CIs. Otherwise,a random effects model was used. The sensitivity of data was analyzed by using the method which reduced the biggest weight of literature. Funnel plot and the Egger’s linear regression method were used to assess publication bias.

No ethics statement to declare.

## Results

### Articles included in the Meta-analysis and study select process

**Fig. 1:**
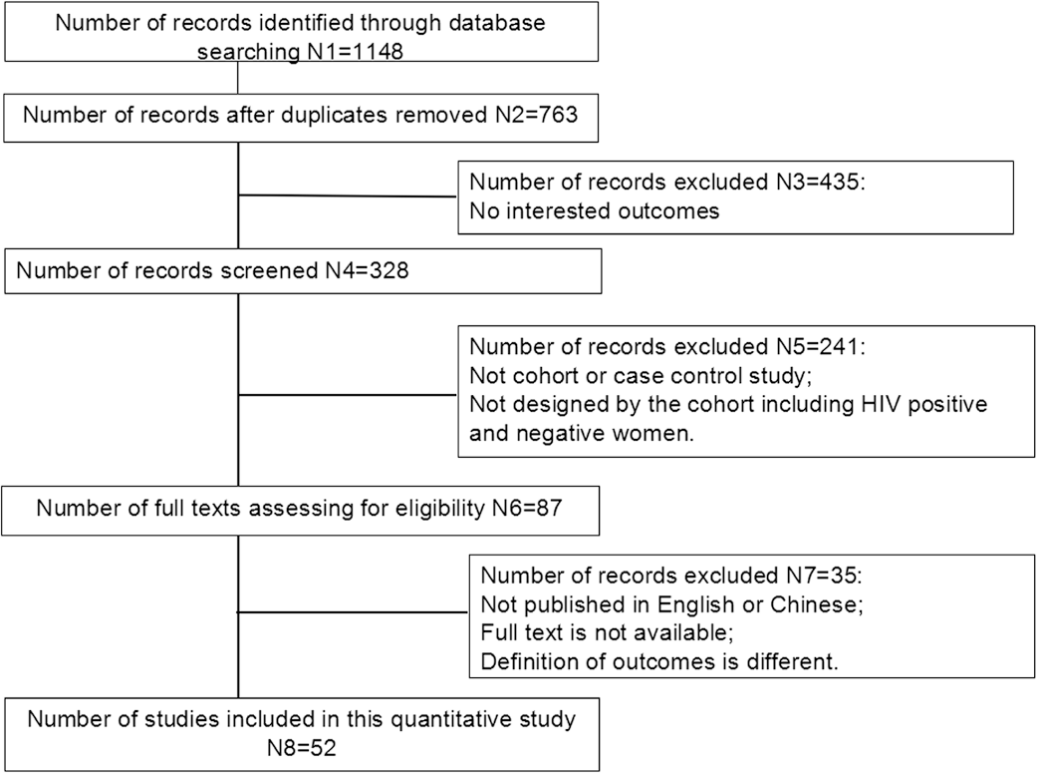
Flow chart of study selection process

A total of 1148 articles were identified. After removing duplicate publications and based on our inclusion and exclusion criteria, 52 articles were included in our analysis (Table 1, Fig. 1) and of them, 43 were related to LBW and 40 were related to PTD. They were all published in English. More than 60 % of the articles were conducted in Africa (*n* = 24) and America (*n* = 11), and 8 were from Europe (*n* = 2) and Asia (*n* = 6).

**Table 1:** Characteristics of the included studies

First author, publication year	Study year	Design type	Study location	Low birth weight			Preterm delivery			Q^a^	Confounding	Exposure to ARVs^*^
				Number of infected women	Rate	OR	Number of infected women	Rate	OR			
Hira et al., 1989 [30]	1987	Prospective	Zambia	227	9.7	3.75	227	6.6	1.37	5	No	No
Lallemant et al., 1989 [31]	1987–1988	Prospective	Congo	64	26.0	3.22				3	No	No
Selwyn et al., 1989 [32]	1989	Prospective	USA				25	32.0	1.01	0	No	No
Braddick et al., 1990 [33]	1986–1989	Retrospective	Malawi	167	9.0	3.00	165	16.0	1.50	7	No	No
Semprini et al., 1990 [34]	1985-	Prospective	Italy				74	15.0	0.42	5	No	No
Halsey et al., 1990 [35]	1976–1985	Prospective	Haiti	199	18.6	1.78	199	6.9	3.35	0	No	No
Lepage et al., 1991 [36]	1988–1989	Prospective	Rwanda	218	17.2	1.40				9	No	No
Mayers et al., 1991 [37]	1985–1989	Prospective	USA				33	36.0	1.76	6	No	No
Alger et al., 1993 [38]	1987–1991	Prospective	USA	101	27.7	1.05				0	No	No
St Louis et al., 1993 [39]	1989–1990	Prospective	Congo				324	20.4	1.31	0	No	Not
Bulterys et al., 1994 [40]	1989–1992	Prospective	Rwanda	274	7.8	1.79	297	28.0	1.50	9	No	No
Temmerman et al., 1994 [8]	1989–1991	Prospective	Kenya	285	19.6	1.40	315	24.1	1.90	9	No	No
Taha et al., 1995 [41]	before 1995	Prospective	Malawi	663	20.1	2.55				7	No	Not stated
Kumar et al., 1995 [42]	1992–1993	Prospective	India	150	21.6	2.12	150	22.0	3.29	9	No	Not stated
Mauri et al., 1995 [43]	1986–1992	Prospective	Italy				38	16.0	1.91	7	No	Not stated
Bloland et al., 1995 [44]	1987–1990	Prospective	Malawi	92	17.4	1.76	85	6.5	1.51	9	No	No
Markson et al., 1996 [45]	1989–1990	Prospective	USA	772	29.0	2.04				7	Yes	Not stated
Bucceri et al., 1997 [11]	1985–1993	Prospective	Italy	153	32.0	1.20	151	21.0	0.90	3	No	Not stated
Castetbon et al., 1999 [46]	1992–1993	Prospective	Rwanda	177	18.6	2.24	177	21.5	2.03	9	Yes, LBW: aOR = 1.21(0.58–2.56)	Not stated
Coley et al., 2001 [12]	1995–1997	Prospective	Tanzania	433	15.2	1.25	420	27.1	1.11	7	Yes, aOR =1.21 and 1.11for LBW and PTD	Not stated
Ellis et al., 2002 [9]	1988–1995	Retrospective	USA	524	29.4	2.11	524	28.9	1.83	7	Yes, aOR:LBW:1.45(1.14–1.86)PTD:1.32(1.04–1.70)	Not stated
Ticconi et al., 2003 [47]	2000–2001	Prospective	Zimbabwe	82		4.36	82	53.7	3.33	7	Yes, LBW: aOR = 3.16(1.80–5.54); PTD: aOR = 4.10(2.17–7.75)	Not stated
Friis et al., 2004 [48]	1996–1997	Retrospective	Zimbabwe	360	14.7	1.74	360	21.1	1.46	9	Not mentioned	Not stated
van Eijk et al., 2004 [49]	1996–2000	Retrospective	Kenya	641	4.8	1.81	641	8.1	1.16	6	No	Not stated
Boer et al., 2007 [50]	1997–2003	Retrospective	Holland	142	17.0	1.72	143	18.0	2.24	4	Yes	Yes
Schulte et al., 2007 [10]	1989–2004 [10]	Retrospective	USA	1744	26.0	1.45	1614	27.0	1.77	9	Yes, aOR = 1.34 for LBW and aOR = 1.65 for PTD	Yes
Haeri et al., 2008 [51]	before 2008	Retrospective	USA	151	30.0	3.04	151	18.0	1.63	0	Yes (spontaneous PTD aOR = 2.27 (1.2–4.3))	Yes
Mitgitti et al., 2008 [52]	1997–2002	Retrospective	Thailand	266	12.0	1.78	247	8.5	1.23	7	Yes, LBW aOR = 1.98(1.26–3.10)	Not stated
Habib et al., 2008 [53]	1999–2006	Retrospective	Tanzania				434	10.4	1.25	5	Yes	Yes, partly
Ezeaka et al., 2009 [54]	2002–2005	Prospective	Nigeria	220	16.8	3.47				7	No	Not stated
Uneke et al., 2009 [55]	2006	Prospective	Nigeria	3	25.0	3.16				0	No	Not stated
Musana et al., 2009 [56]	2004–2005	Prospective	Kenya	68	56.0	2.80	68	73	2.20	3	No	Not stated
Olagbuji et al., 2010 [57]	2007–2008	Prospective	Nigeria	203	18.2	5.43	203	12.8	1.98	9	No	Yes
Pattinson et al., 2010 [58]	2006–2008	Retrospective	South Africa	3014	19.8	1.47				0	No	Yes, partly
Patil et al., 2011 [13]	2002–2003	Prospective	India	212	31.1	0.77	212	13.7	1.13	7	No	Yes, partly
Asavapiriyanont et al., 2011 [59]	2004–2008	Prospective	USA	420	12.6	0.94				0	Not mentioned	Yes, partly
Ndirangu et al., 2012 [60]	2001–2004	Prospective	South Africa	1189	9.8	1.48	1189	21.8	1.39	9	Yes, aOR for PTD = 1.07	No
Lopez et al., 2012 [61]	1986–2010	Retrospective	Spain				519	19.7	2.60	9	Yes, aOR = 2.5(1.9–3.5)	Yes, partly
Chen et al., 2012 [62]	2009–2011	Retrospective	Botswana				9504	23.7	1.50	4	Yes, aOR = 1.3(1.3–1.4)	Yes
Nkhoma et al., 2012 [63]	2005–2006	Prospective	Malawi	45	26.7	2.32				4	Yes, aOR = 3.08(1.40–6.79)	Yes
Muhangi et al., 2013 [29]	2003–2005	Prospective	Uganda	121	10.0	1.80				3	No	Yes, only 5
Ezechi et al., 2013 [64]	2004–2011	Prospective	Nigeria	2158	9.4	3.01	2105	13.1	2.10	9	Yes, aOR = 2.95(1.95–3.10); 2.05(1.3–3.1)	Yes
Duan et al., 2014 [65]	2010–2013	Retrospective	China	300	14.7	6.27	300	10.3	1.81	3	No	Yes
He et al., 2013 [66]	2007–2009	Retrospective	China	58	3.4	0.65	58	5.2	1.53	7		Yes
Wang et al., 2009 [67]	2007–2008	Retrospective	China	35	14.3	3.55				3	No	Yes
Han et al., 2004 [68]	1997–1999	Case control	Tanzania	37	18.9	4.43	37	16.2	3.68	3	No	Not stated
Dong et al., 2001 [69]	1995–1999	Case control	Tanzania	86	16.3	2.40	86	14.0	3.08	3	No	Not stated
Macdonald et al., 2015 [70]	2002–2003, 2010–2011	Retrospective	Canada	615	12.5	1.90	615	14.6	1.76	7	Yes	Yes
Salihu et al., 2013 [71]	1998–2007	Prospective	USA	4634		1.73	4634		1.35	9	Yes	Not stated
Boyajian et al., 2012 [72]	2003–2010	Retrospective	Canada	91	20.2	2.91	91	15.6	1.70	7	Yes	Yes
Brown et al., 2012 [73]	2000–2008	Retrospective	USA	71	43.7	4.23	71	40.9	2.83	6	No	Yes
Da Costa et al., 2013 [74]	1995–2005	Prospective	Brazil				713	15.0	1.57	6	Yes, aOR 1.26(0.90–1.770	Not stated

*CI* confidence interval, *LBW* low birth weight; *OR* odds ratio, *aOR* adjusted odds ratio, *PTD* preterm delivery

^a^quality score of articles calculated by Newcastle-Ottawa Scale; ARVs* antiretroviral drugs exposure before or during pregnancy

The prevalence of infants born before 37 weeks’ gestation ranged from 5.2 to 73.0 % in HIV infected women and 2.2 to 32 % in HIV uninfected women. The prevalence of LBW among HIV infected women ranged from 3.4 to 56.0 % and 2.5 to 36.9 % in HIV uninfected women. Odds ratios for the association of HIV exposure ranged from 0.65 to 6.27 with LBW and ranged from 0.42 to 3.68 with PTD.

### Impact of maternal HIV infection on LBW and PTD

**Fig. 2:**
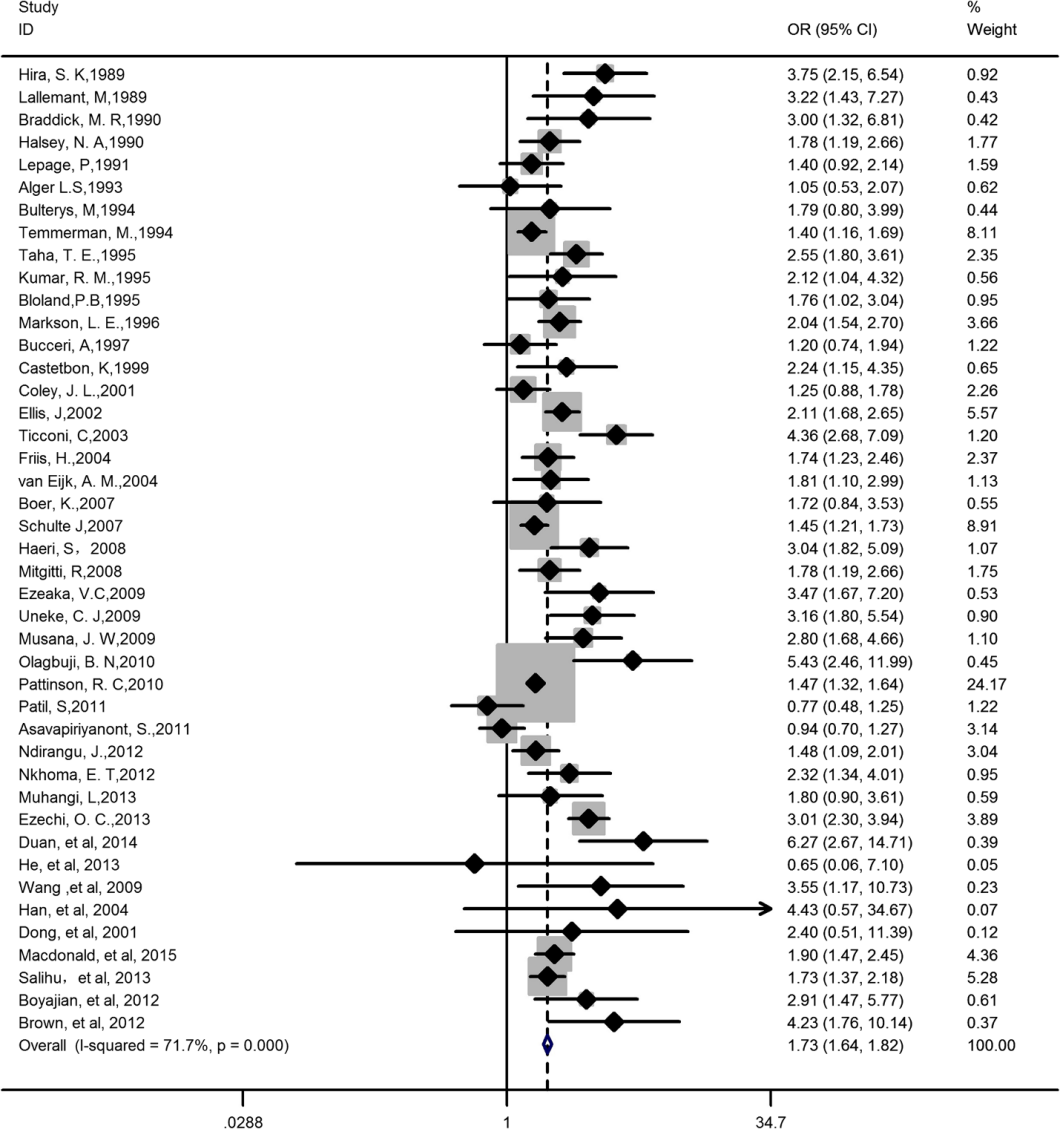
Forest plot of odds ratio for low birth weight. Pooled OR = 1.73, 95 % CI: 1.64, 1.82; Heterogeneity chi-squared = 148.65 (d.f. = 42), *p* = 0.000, I-squared (variation in ES attributable to heterogeneity) = 71.7 %; Test of ES = 1: z = 20.06, *p* = 0.000

**Fig. 3:**
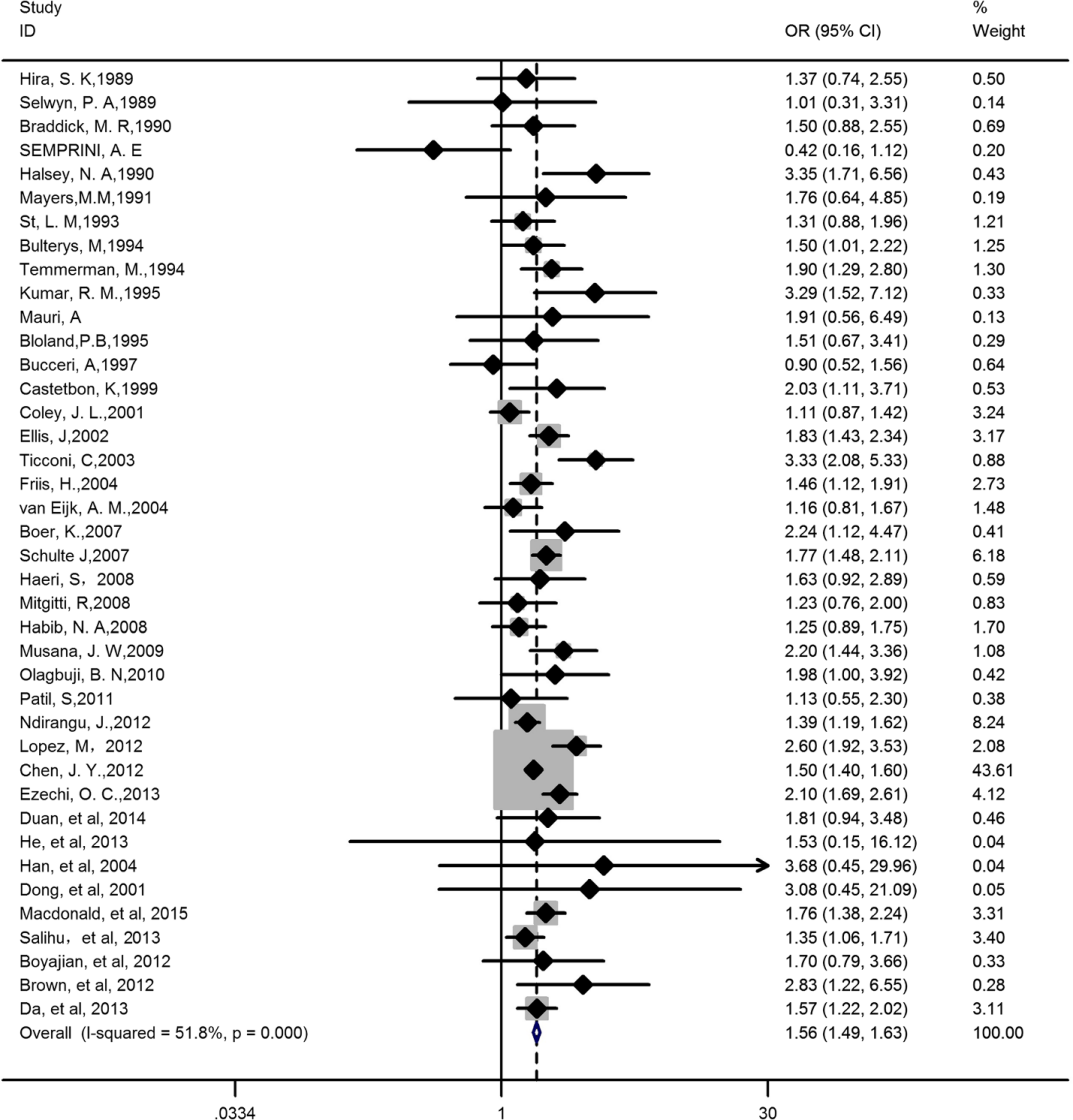
Forest plot of odds ratio for preterm delivery. Pooled OR = 1.56, 95 % CI: 1.49, 1.63; Heterogeneity chi-squared = 80.95 (d.f. = 39), *p* = 0.000, I-squared (variation in ES attributable to heterogeneity) = 51.8 %; Test of ES = 1: z = 19.79, *p* = 0.000

There was significant heterogeneity among studies for maternal HIV infection associated with LBW/PTD (I^2^ = 71.7 %, *P* < 0.05, and I^2^ = 51.8 %, *P* < 0.05 for LBW and PTD, respectively) suggesting that the summary measures need to be interpreted with caution. Pooled ORs for LBW and PTD from random effect models were presented in Figs. 2 and 3, respectively. The summary OR was 1.73 (95 % CI: 1.64, 1.82, *P* < 0.001) for LBW and 1.56 (95 % CI: 1.49, 1.63) for PTD, indicating that HIV infected women had approximately 2-fold risk to deliver low birth weight or preterm babies compared with uninfected ones.

### Test for publication bias

Begg’s and Egger’s Tests suggested that there were significant publication biases among the 43 studies of LBW (Begg’s Test: z = 2.15, *P* = 0.03; Egger’s Test: *t* = 3.53, *P* = 0.001) (Additional file 1). After adjusted by using the Trim and Filling method, the pooled OR was 1.77 (95 % CI: 1.57, 2.00), which was near to the original OR (1.73, 95 % CI: 1.64, 1.82). No significant publication bias was detected for the 40 articles of PTD (Begg’s Test: z = 0.79, P = 0.43; Egger’s Test: t = 1.69, *P* = 0.10) (Additional file 2).

### Sensitivity analysis

After removing the article that counted the biggest weight, there was no significant change of the results. The pooled ORs were 1.98 (95 % CI: 1.76, 2.23) for LBW and 1.62 (95 % CI: 1.49, 1.77) for PTD, respectively. After stratified by location of study population, the significant associations between maternal HIV infection and LBW or PTD were still found in the studies from Africa, America and Asia, except those from Europe. Studies from Africa (OR = 2.18, 95 % CI: 1.84, 2.59), America (OR = 1.80, 95 % CI: 1.49, 2.19) and Asia (OR = 1.99, 95 %CI: 1.05, 3.74) showed significant pooled ORs for LBW, and for PTD the corresponding pooled ORs were 1.56 (95 % CI: 1.40, 1.75), 1.69 (95 % CI: 1.53, 1.88) and 1.60 (95 % CI: 1.10, 2.35), respectively. These associations were all significant in the two groups of developing and developed countries. No significant difference in the relationship was detected across study periods and their pooled ORs were all statistically significant for both LBW and PTD. But studies during 2005–2015 were at slightly higher risk for both LBW and PTD compared with the other two groups (Table 2).

**Table 2:** Results of subgroup analysis

Factors	Groups	Low birth weight				Preterm delivery			
		No. of studies	OR (95 % CI)	*P*	*I*^*2*^ (%)	No. of studies	OR (95 % CI)	*P*	*I* ^*2*^
Design type	Prospective	27	1.93(1.64, 2.28)	<0.001	75.8	23	1.60(1.39, 1.84)	<0.001	59.8
	Retrospective	16	2.05(1.72, 2.45)	<0.001	62.2	17	1.67(1.49, 1.86)	<0.001	39.5
Study period	Before 1995	15	1.90(1.61, 2.25)	<0.001	54.4	15	1.59(1.31, 1.93)	<0.001	41.2
	1995–2004	14	1.72(1.42, 2.08)	<0.001	63.1	15	1.55(1.34, 1.80)	<0.001	66.1
	2005–2015	14	2.52(1.90, 3.33)	<0.001	83.7	10	1.79(1.55, 2.06)	<0.001	37.9
Study location	Africa	24	2.18(1.84, 2.59)	<0.001	73.5	19	1.56(1.40, 1.75)	<0.001	52.6
	America	11	1.80(1.49, 2.19)	<0.001	73.1	11	1.69(1.53, 1.88)	<0.001	6.2
	Europe	2	1.34(0.90, 2.00)^a,^	0.15	0	5	1.40(0.73, 2.68)^a^	0.32	80.4
	Asia	6	1.99(1.05, 3.74)	0.03	77.3	5	1.60(1.10, 2.35)	0.02	27.3
Country economic	Developing	31	2.12(1.81, 2.48)	<0.001	72.5	26	1.60(1.44, 1.76)	<0.001	48.9
	Developed	12	1.75(1.44, 2.12)	<0.001	71.9	14	1.67(1.41, 1.98)	<0.001	52.5
ARVs exposure	No	10	1.75(1.42, 2.17)	<0.001	51.7	9	1.54(1.23, 1.92)	<0.001	44.5
	Not stated	17	2.07(1.78, 2.42)	<0.001	51.2	17	1.55(1.32, 1.82)	<0.001	56.2
	Yes	16	2.04(1.61, 2.57)	<0.001	82.1	14	1.77(1.55, 2.02)	<0.001	52.4
Overall		43	1.98(1.76, 2.23)	<0.001	71.7	40	1.62(1.49, 1.77)	<0.001	51.8

*No* number, *CI* confidence interval, *ARVs* antiretroviral drugs, *OR* odds ratio

^a^No significant difference/no statistical significance (*P* > 0.05)

We also conducted a subgroup analysis based on the information of antiretroviral drugs (ARVs) usage. ARVs usage before or during pregnancy did not decrease the risks of both LBW and PTD. Women who took ARVs were at similar risk of delivering low birth weight infants (OR = 2.04, 95 % CI: 1.61, 2.57) compared with those did not (OR = 1.75, 95 % CI: 1.42, 2.17). For PTD, the pooled ORs were 1.77 (95 CI: 1.55, 2.02) for women who took drugs, 1.54 (95 % CI: 1.23, 1.92) for those who did not took drug and 1.55 (95 % CI: 1.32, 1.82) for those who provided no information about AVRs usage (Table 2).

## Discussion

It has been previously reported that compared with unexposed children, children who encountered intrauterine HIV exposure are more vulnerable to stunting, underweight and wasting, and their birth weight, height and head circumference are generally lower than their unexposed counterparts [15]. However, it was controversial for the effect of maternal HIV infection on both LBW and PTD. Brocklehurst et al. [5] reported that the increasing risks of LBW and PTD were associated with maternal HIV infection. And they found that women in developing countries had higher risks of both LBW and PTD than those in developed countries. Our study, which included a number of newer studies in the meta-analysis also indicated that maternal HIV infection increased the risks of both LBW and PTD. We found that these associations were not affected by different study periods or antiretroviral drugs usage. In addition, we did not find marked difference in these associations between developing and developed countries.

The HIV associated LBW and PTD might be related to the damaged human immune system, especially the reduced CD4+ T cells and immunosuppression. Previous studies documented that women with CD4 cell counts <350 cells/mm^3^ had an increased risk of having LBW infants (RR = 1.57; 95 % CI: 1.16, 2.12) compared to women with higher CD4 cell counts [16, 17]. There is a possibility that women are immunocompromised during pregnancy [18], and if accompanied with HIV infection, disease progress might be accelerated. Simultaneously, reproductive tract infections, which are contributed to the incidence of adverse pregnancy outcomes, would occur more frequently due to the immunosuppression [19]. Some studies have reported that HIV-1 can replicate in the placenta [20], and it has also been shown that HIV-1 infection may alter the cytokine profile in the placenta [21, 22]. This may affect the function of placenta during pregnancy, and then restrict the development of fetal, which might be another incentive of LBW and PTD.

After stratified by antiretroviral drugs use before or during pregnancy, we found that HAART or other regimens of antiretroviral therapy (ART) had no obvious effect on the associations between maternal HIV infection and LBW/PTD. It is suggested that intrauterine ARVs exposure did not decrease or increase the risk of LBW or PTD in HIV infected women. And this is consistent with the findings reported by van der Merwe et al. and Townsend et al. [3, 4]. However, Papp et al. [23] suggested that Protease Inhibitor (PI)-based ART could increase the risk of adverse pregnancy outcomes mainly due to lower level of progesterone, which was significantly associated with fetal weight. Sibude et al. [24] also found that ARVs and, particularly, with the initiation of ritonavir-boosted PI therapy during pregnancy were correlated with PTD in HIV infected women. Though we did not found such effect of ARVs on the association between maternal HIV infection and LBW/PTD, no details of information for AVRs and therapy regimens may contribute to this. We found that studies during 2005–2015 were at slightly higher risk for both LBW and PTD compared with the other two groups, it might be related with the increasing use of combination ART earlier in pregnancy, and more use of PI for ART in recent years. Kourtis et al. [25] had found that use of PI in ART may increase the risk of PTD compared with ART without PI. And different initiation time of ART have different influences on the risk of PTD. Townsend et al. [26] also indicated that HARRT was associated with PTD. We have ever tried to analyze the data by the information of ART regimens, initiation time, ARVs duration, etc., however, there were not enough information to support the analysis of the data after stratification, for the information of ART were not provided or not detailed described in all articles. Thus we could not know if there any influences of different ART regimens or different initiation time on the association between maternal HIV infection and LBW/PTD. On the other hand, ARVs might be responsible for adverse pregnancy outcomes such as LBW and PTD, but its high effectiveness in the prevention for mother to child transmission outweighed its risk of LBW/PTD suggested by Santini et al. [27].

Maternal HIV infection was found to be related with both LBW and PTD in some places such as Africa and America. Moreover, no difference in the relationship was detected among the different study periods. This, to a certain degree, reflected the non-ideal treatment and health care development in reducing the risk of LBW and PTD for HIV infected women. So we look forward to finding new regimens or methods combined with other measurements like perinatal health care for pregnant women to decrease the risk of adverse pregnancy outcomes.

There are several limitations to note. Our meta-analysis dataset was not complete due to some restrictions of accessing to full texts. Another limitation is that we could not determine if maternal HIV infection had direct effects on LBW/PTD. We could not rule out the possibility that the association is secondary to HIV-associated deficiency, for example maternal nutrition deficiency, or other risk factors of adverse pregnancy, such as drug use, smoking, etc. And we could not get the association between maternal HIV infection and LBW/PTD after controlling for different clinical stages of HIV infection in this analysis because of the absence of such information. The relationship between maternal HIV infection and LBW/PTD is likely affected by the clinical stage of HIV infection. Study conducted by Coley et al. in Tanzania [28] indicated that although HIV infected asymptomatic women did not have a higher risk of having LBW infants compared with uninfected women (OR = 1.25; 95 % CI: 0.88, 1.79), symptomatic HIV-infected women who were in Stage 2 or higher according to the WHO staging system had about 2-time higher risks for low birth weight (RR = 2.29; 95 % CI:1.34, 3.92) and prematurity (RR = 1.93; 95 % CI: 1.35, 2.77) compared with HIV-uninfected ones. This is consistent with the finding of Bucceri et al. [11] and Muhangi et al. [29]. Publication bias is always a concern for systematic reviews. Publication bias in this meta-analysis was analyzed by using funnel plot and the Begg’s and Egger’s test methods. Significant publication bias was detected among studies of LBW and proper adjustment was conducted. And we found the results of this analysis had good stability by sensitivity analysis.

## Conclusions

In conclusion, the findings of this study indicated that maternal HIV infection was associated with increased risks of both low birth weight and preterm delivery. However, the associations were moderate. No difference in the relationships between maternal HIV infection and adverse pregnancy outcomes was detected among different study periods. ARVs were not found to decrease risks of either low birth weight or preterm delivery associated with maternal HIV exposure.

## Additional files

Additional file 1: **Funnel plot of publication bias for low birth weight.** (TIFF 124 kb)
Additional file 2: **Funnel plot of publication bias for preterm delivery.** (TIFF 127 kb)

## Abbreviations

HIV: Human immunodeficiency virus
LBW: Low birth weight
PTD: Preterm delivery
HAART: Highly active antiretroviral therapy
MTCT: Mother-to-child transmission
ARVs: Antiretroviral drugs
PI: Protease inhibitor
