# Supplementary figures and images for: Association between maternal HIV infection and low birth weight and prematurity: a meta-analysis of cohort studies
Source: BMC Pregnancy Childbirth. 2015 Oct 8;15:246. doi: 10.1186/s12884-015-0684-z (PMC4599647; doi:10.1186/s12884-015-0684-z)

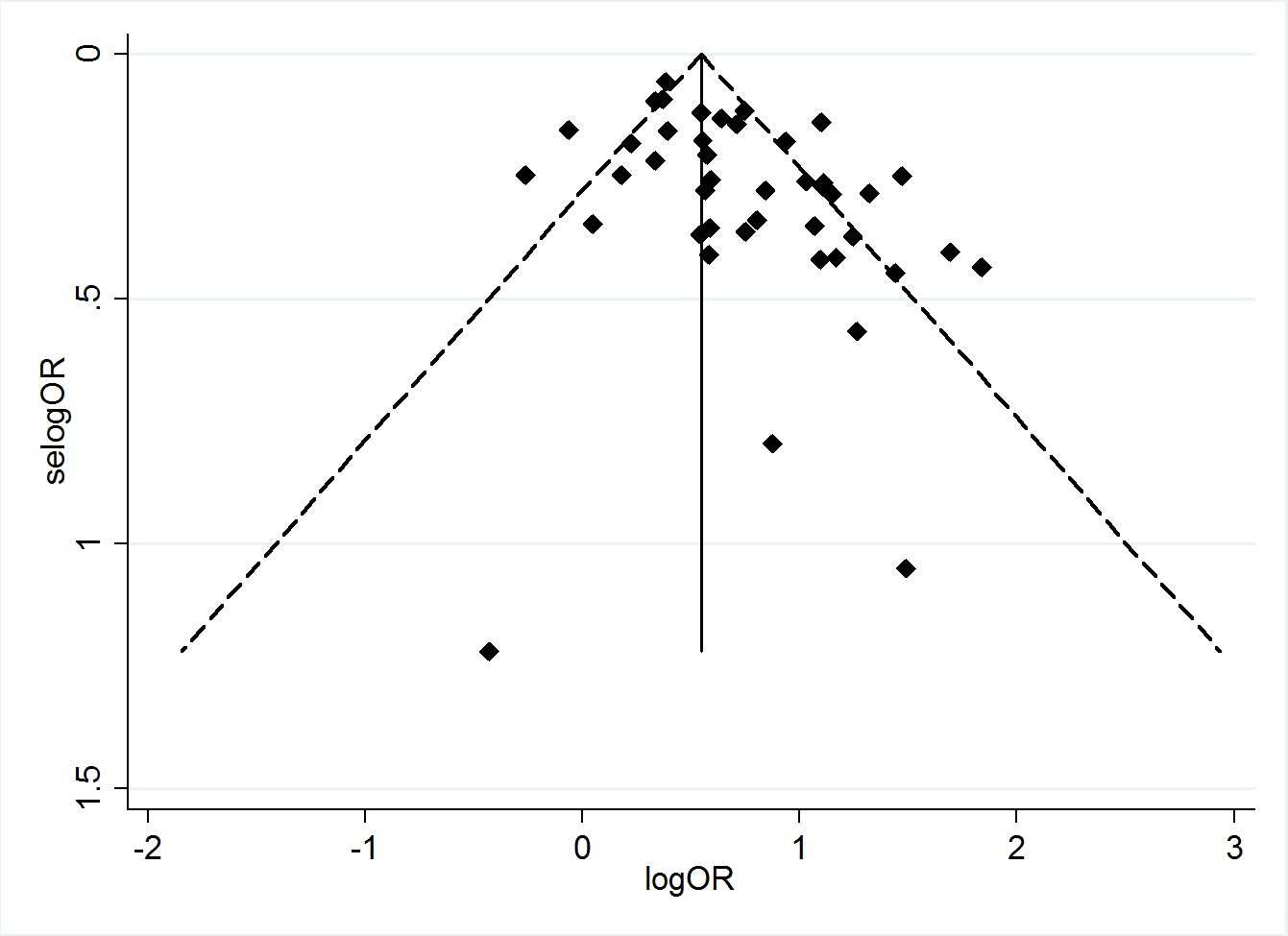

Supplement: Additional file 1: — Funnel plot of publication bias for low birth weight. (TIFF 124 kb) [file 12884_2015_684_MOESM1_ESM.tiff]

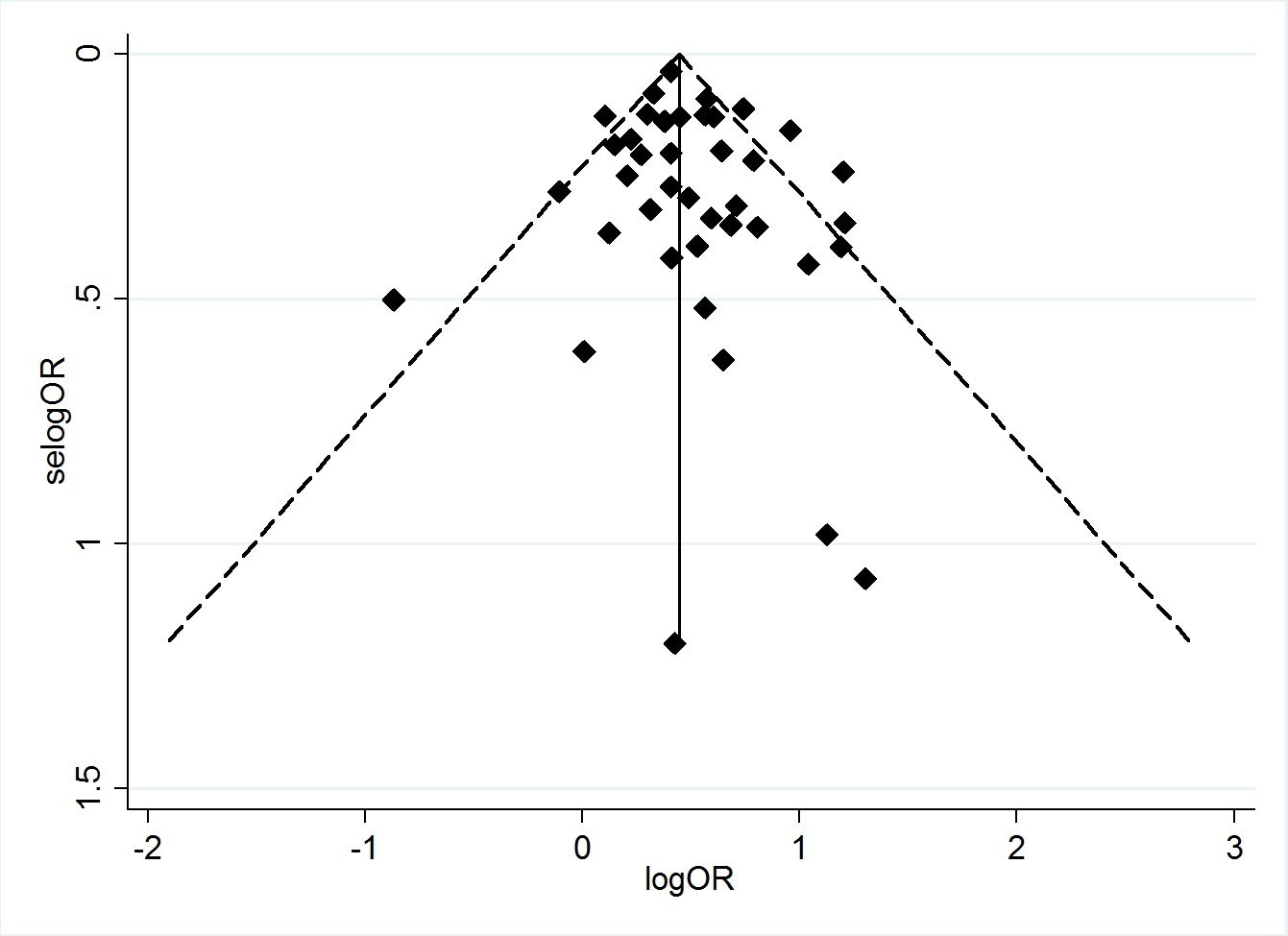

Supplement: Additional file 2: — Funnel plot of publication bias for preterm delivery. (TIFF 127 kb) [file 12884_2015_684_MOESM2_ESM.tiff]
